# A benchmark for domain adaptation and generalization in smartphone-based human activity recognition

**DOI:** 10.1038/s41597-024-03951-4

**Published:** 2024-11-02

**Authors:** Otávio Napoli, Dami Duarte, Patrick Alves, Darlinne Hubert Palo Soto, Henrique Evangelista de Oliveira, Anderson Rocha, Levy Boccato, Edson Borin

**Affiliations:** 1 Institute of Computing, Unicamp, Brazil; 2 School of Electrical and Computer Engineering, Unicamp, Brazil

**Keywords:** Computer science, Scientific data

## Abstract

Human activity recognition (HAR) using smartphone inertial sensors, like accelerometers and gyroscopes, enhances smartphones’ adaptability and user experience. Data distribution from these sensors is affected by several factors including sensor hardware, software, device placement, user demographics, terrain, and more. Most datasets focus on providing variability in user and (sometimes) device placement, limiting domain adaptation and generalization studies. Consequently, models trained on one dataset often perform poorly on others. Despite many publicly available HAR datasets, cross-dataset generalization remains challenging due to data format incompatibilities, such as differences in measurement units, sampling rates, and label encoding. Hence, we introduce the DAGHAR benchmark, a curated collection of datasets for domain adaptation and generalization studies in smartphone-based HAR. We standardized six datasets in terms of accelerometer units, sampling rate, gravity component, activity labels, user partitioning, and time window size, removing trivial biases while preserving intrinsic differences. This enables controlled evaluation of model generalization capabilities. Additionally, we provide baseline performance metrics from state-of-the-art machine learning models, crucial for comprehensive evaluations of generalization in HAR tasks.

## Background & Summary

In recent years, there has been an increasing interest in expanding smartphones’ capabilities so that they can detect user, system, and environmental patterns and autonomously take actions to assist the user, thus providing an adaptable and customized experience/interaction. For instance, the smartphone can detect that the user is driving and automatically switch to a driving mode, or detect that the user is walking and automatically switch to a fitness mode.

Human activity recognition (HAR) using smartphone inertial sensors constitutes one example of such efforts, which involves the automatic identification of what the user is doing based on samples of different sensors, such as accelerometer (henceforth denoted as Acc) and gyroscope (Gyr)^1^. In particular, inertial sensors have proved to be useful in discriminating among different activities and other related tasks, allowing for the development of applications in several domains^2–15^.

However, a major challenge in HAR is the generalization of models to new scenarios, a process known as domain adaptation or domain generalization in machine learning^16,17^, which involves transferring knowledge from a source domain to a target domain, where the source and target domains have different distributions. These differences can arise from several factors, including: (i) sensor differences, such as the type and quality of sensors and the sampling rate, which can affect data quality and lead to variations in resolution and granularity; (ii) sensor placement, such as body position (e.g., wrist, waist, ankle) and attachment method (e.g., tightly strapped, loosely worn), which can influence movement patterns and capture different motion patterns for the same activity; (iii) data collection protocols can vary due to environmental conditions (indoor vs. outdoor, different weather conditions) and activity execution, with differences in how activities are performed (e.g., walking speed, running style) resulting in variations in recorded data; (iv) user demographics such as age, gender, height, weight, and physical fitness, as well as the number of users in the dataset, are crucial for generalizing to a broader population; and (v) device differences, including hardware variations and preprocessing techniques such as filtering, smoothing, and feature extraction methods, also impact data quality and the information extracted.

In HAR literature, several datasets have been created to allow the evaluation of HAR models, typically used in isolation^3,18–20^. Some datasets are extensive and encompass a wide range of activities collected in real-world scenarios, while others are smaller and feature a limited number of activities in controlled environments. These datasets include various users, activities, and sensors, providing a good starting point for developing HAR models. For example, the RealWorld dataset^3^ allows for studying the impact of sensor position on model performance in a controlled setting. At the same time, the ExtraSensory^20^ dataset facilitates the study of data collection protocol impacts on model performance in real-world environments.

Even though these datasets present good variability, it is important to notice that each dataset constitutes a domain due to bias introduced by data collection protocols, demographic settings, and other factors. Thus, it is essential to evaluate the generalization capabilities of models across different datasets to ensure that they perform well in new scenarios, something often overlooked in the literature. This is a very challenging task partly due to the lack of a standardized approach for evaluating model generalization among different datasets, as they have different unit measurements, sampling rates, gravity components, as well as tasks and labels that are not shared among them, making it difficult to assess the generalization capabilities of models across different datasets.

We present the DAGHAR benchmark^21^, a curated dataset collection designed for domain adaptation and domain generalization studies in HAR tasks. It features raw inertial sensor data sourced exclusively from smartphones. We carefully selected six publicly available datasets and standardized them for accelerometer units of measurement, sampling rate, gravity component, activity labels, user partitioning, and time window size. This standardization process allowed us to create a comprehensive benchmark for evaluating the generalization capabilities of HAR models in cross-dataset scenarios.

We demonstrate that the standardization process does not remove the intrinsic differences among the datasets, but it enables a more controlled evaluation of model generalization capabilities. To accomplish this, we also provide a set of baseline performance metrics from state-of-the-art deep learning models and classical machine learning models applied to the DAGHAR benchmark^21^. This is a crucial first step towards creating a more comprehensive benchmark for HAR.

## Methods

Here, we describe the datasets selected to compose the DAGHAR benchmark^21^ and the preprocessing steps applied to standardize each dataset. Using the *t*-SNE^22^ visualization, we show the data distribution of the datasets in the time and frequency domains, and we also report a performance evaluation of state-of-the-art deep learning and classical machine learning models on the standardized views of the datasets to verify that the proposed standardization does not remove the intrinsic differences among the datasets.

### Datasets selection

Human activity recognition (HAR) is a well-established field of machine learning and signal processing research. Various approaches can be employed to tackle the HAR task, including wearable sensors, cameras, or smartphones. This study focuses on non-invasive continuous monitoring sensors, narrowing our scope to inertial sensors, specifically tri-axial accelerometers and tri-axial gyroscope sensors, commonly found in smartphone and wearable inertial measurement units (IMUs). Additionally, we concentrate on smartphone sensors due to their widespread availability and accessibility to the general public, unlike wearable sensors, which are less common. Consequently, our search for HAR datasets specifically targeted those containing data from smartphone inertial sensors.

The set of publicly available datasets that meet this requirement and are known by the HAR literature is limited. To address this, we conducted an extensive survey of over 40 HAR datasets, gathering key information such as the number of samples, types of activities recorded, participant demographics, sampling rates, sensor characteristics, citation frequency, and data collection protocols. From this initial list, we selected datasets based on the following criteria: (**CR1**) the availability of raw data, not just preprocessed data, to ensure flexibility in analysis and preprocessing; (**CR2**) data integrity, where the dataset must: (i) include timestamps, (ii) be free from missing values, inconsistencies, or irregularities, (iii) have at least one associated research publication, (iv) originate from smartphones, and (v) include both accelerometer and gyroscope data. These sub-criteria are essential to ensure the data’s reliability and its suitability for further analysis; datasets failing any sub-criteria were excluded; (**CR3**) the inclusion of a substantial set of shared activities, ensuring that the dataset can support a broad range of activity recognition tasks; (**CR4**) a focus on regular human daily activities, excluding datasets that primarily feature sports, geographic-based activities, or require invasive data collection methods, such as using microphone data. Table 1 shows the datasets analyzed according to these criteria.

**Table 1:** List of datasets found in literature and their respective criteria status.

Dataset	CR1	CR2	CR3	CR4
KuHar^23^	✓	✓	✓	✓
MotionSense^24^	✓	✓	✓	✓
RealWorld^3^	✓	✓	✓	✓
UCI^25^	✓	✓	✓	✓
WISDM^2^	✓	✓	✓	✓
RealLifeHAR^36^	✓	✓		✓
The SHL Dataset^37^	✓	✓		✓
HARSENSE^38^	✓		✓	✓
TNDA-HAR^39^	✓		✓	✓
Extrasensory^20^	✓		✓	✓
CHARM^26^	✓		✓	✓
FallDAD^40^*	✓		✓	✓
HASC-Challenge^41^	✓		✓	✓
PAR^42^	✓		✓	✓
Opportunity^43^	✓		✓	✓
Bike&Safe^44^*	✓			✓
DRIVER/DRIVER-21^45^*	✓			
Phone-Sensor-Driving^46^*	✓			
Writing-Behavior^47^*	✓			
TOOTHBRUSHING^48^	✓			
eGLASSES^49^	✓			
Entry-Exit-CAR^50^	✓			
RecodGait v1^51^	✓			
RecodGait v2^8^	✓			
DailySports^52^		✓	✓	✓
LAR^53^		✓	✓	✓
FallAllD^54^			✓	✓
HAD-AW^55^			✓	✓
DU-MD^56^			✓	✓
Crowds^57^				
Embedded^58^				
INDOOR^59^*				
Parkinson-acoustic^60^*				
Distributed-Recognition^61^				
ElderlyFall^62^				
FallADL^63^				
HealthDetection*				
Mob-Battery-20*				
UbiqLog^64^				
WIDAR^65^				
HAR-AUDIO^66^				

**CR1**: Availability of raw data;

**CR2**: Data integrity;

**CR3**: Inclusion of a substantial set of shared activities;

**CR4**: Regular human daily activities.

Asterisks means that dataset has no published work associated.

These criteria allowed us to select three datasets, which we describe in the sequence. Initially, we selected only datasets containing at least the activities: sit, stand, walk, walk upstairs, walk downstairs, and run. However, to increase the number of datasets in our experiments, we allowed datasets with at least four out of the six activities, so the WISDM and the UCI-HAR datasets were incorporated.

After analyzing the datasets, we decided to work with: KU-HAR version 5 (raw time domain data folder)^23^, MotionSense^24^, RealWorld^3^, WISDM^2^, and the updated version of the UCI-HAR dataset^25^. Table 2 summarizes the main characteristics of these datasets. Notice that they differ regarding the smartphone position, the metric used to record the accelerometer samples, the sampling rate, and the number of users and activities registered.

**Table 2:** Selected datasets and their main features.

Acronym	Dataset Name	Smartphone Position	Accelerometer Metric^b^	Sampling Rate	Number of Users/Activ
KH	KU-HAR^23^	waist bag	m/s^2^	100 Hz	90/18
MS	MotionSense^24^	pocket	G	50 Hz	24/6
RW	RealWorld^3^	thigh/waista	m/s^2^	50 Hz	15/8
UCI	UCI-HAR^25^	waist bag	G	50 Hz	30/5
WDM	WISDM^2^	pocket	m/s^2^	20 Hz	51/18

^a^This dataset employs multiple smartphones placed in different locations during data collection (*e.g*., thigh, waist, shin, head, *etc*.). We used data collected by sensors placed at thigh (RW-Thigh) and waist (RW-Waist), which should be equivalent to a pocket and a waist bag.

^b^All datasets record gyroscope samples in rad/s.

KU-HAR and WISDM contain samples for 18 different human activities, while the other datasets contain at most eight different activities. We kept only the samples from our standard activity set, aforementioned, and discarded the remaining activities. Table 3 shows the set of activities kept for each dataset.

**Table 3:** Set of activities selected per dataset.

Activity	KH	MS	RW	WISDM	UCI
Sit	✓	✓	✓	✓	✓
Stand	✓	✓	✓	✓	✓
Walk	✓	✓	✓	✓	✓
Upstairs	✓	✓	✓		✓
Downstairs	✓	✓	✓		✓
Run	✓	✓	✓	✓	

We decided to discard some well-known datasets from our analysis, but they are worth mentioning. The first one is the CHARM dataset^26^. At first glance, the CHARM dataset would easily fit our criteria, but we spotted a crucial problem with the gyroscope data at the preprocessing stage. After analyzing a significant portion of the Acc and Gyr time series data, we found that the Gyr samples in CHARM correspond to slightly distorted versions of the Acc samples (delayed signal plus noise). Moreover, we verified that the signals in CHARM display significant irregularities in the sampling rate.

Another dataset worth mentioning is the ExtraSensory dataset^20^, probably the largest publicly available dataset. One of its main characteristics is the data collection protocol, called by the authors “in-the-wild”: data collected from users engaged in their regular natural behavior. Users were responsible for labeling the collected data as recorded or later. This option led to a huge imbalance in the number of samples per activity and a certain distrust of the data labels. So, we decided to reserve the ExtraSensory dataset for future explorations with themes that might better suit its characteristics.

Once the data was selected, we created *views* of the dataset. These *views* represent the dataset as a pair (**X,** *y*), where **X** is a matrix with *N* samples, each with dimension *d*, and *y* is the corresponding set of labels. The views are obtained after preprocessing the raw data files. Each dataset sample may vary from a few seconds to a few minutes of inertial sensor recordings. We sliced the original time series of each Acc / Gyr axis into non-overlapping 3-second windows. Although there are several works that used the fixed duration windowing scheme with values as small as 0.5 seconds to values larger than 30 seconds^2,3,24,27^, the most common range for HAR tasks is between 1–5 seconds, as described in detail in the systematic review work by Straczkiewicz *et al*.^1^. More specifically, according to Wang *et al*.^28^, a window duration between 2.5–3.5 seconds allows a better balance between performance and latency for human activity recognition tasks.

It was necessary to treat the irregular time spacing between consecutive observations within each time series in order to produce the initial views of the datasets. Since UCI and MotionSense do not provide timestamp annotations, we relied on the authors’ sampling rate description and assumed uniform sampling.

We could analyze the sampling period in more detail as KU-HAR, WISDM, and RealWorld include the timestamp annotation. In the case of RealWorld, according to the authors, the sampling rate was 50 Hz. However, we verified that a non-negligible amount of data was inconsistent with this value, which indicates that some distortion occurred during the recording. By carefully analyzing these samples, we observed that the distortion probably lies in the timestamps. If they were discarded and regular sampling with 50 Hz was assumed, the distorted samples presented a consistent behavior compared to those with more regular sampling. This observation raises some questions regarding the reliability of the timestamp annotation, such as: What kind of distortions can occur in these sensors? What are the reasons behind such distortions? How can we mitigate their occurrence?

For the WISDM dataset, the authors indicated a 20 Hz sampling rate with the following remark: “*Due to the nature of the Android OS, the sampling rate is only taken as a suggestion, so actual sampling rates sometimes differed*”^2^. In our analysis, we could verify a trimodal distribution of the instantaneous sampling rate centered at 50 Hz, 25 Hz, and 20 Hz, with less than half of the samples matching the nominal sampling rate. This discrepancy may be due to the differences in the devices used during the data recording (three smartphone models were used in WISDM). However, this information cannot be confirmed since it is not available. Thus, we decided to interpolate the data using the cubic spline method^29^ to regularize the sampling rate to 20 Hz.

Another relevant aspect to observe is the presence or absence of the gravitational contribution in the accelerometer time series. Let us first define three perspectives of the Acc signal: the body acceleration (the component of the sensor’s movement), the gravity component (which affects all Acc axes during rotational motion), and the total acceleration (body plus gravity). The Acc can only sense the total acceleration, so some procedures must be performed to separate the body and gravity acceleration. Several methods described in the literature are suitable for this task. However, the most common involves applying a high-pass Butterworth filter of low order (e.g., order 3) with a cutoff frequency below 1 Hz^1^. Similarly to the previous observations concerning the sampling rate, the datasets are significantly diverse in terms of the set of acceleration series each one provides: some datasets provide only the body acceleration or the total acceleration, body and gravity acceleration, body and total acceleration, or even gravity and total acceleration. Additionally, sometimes the process of capturing body and gravity acceleration is reported, and sometimes, not. We decided to maintain all the natural (innate) aspects of the data. For the datasets that provide only body acceleration (KuHar and UCI), we process the data without adding gravity. For the MotionSense dataset, that provide body and gravity acceleration separately, we sum both signals. For the datasets that provide the total acceleration, we use this signal directly.

In the literature, several preprocessing steps have been explored, among which we cite^20^: denoising (signals are filtered out from unnecessary or redundant information); rotation (signals are projected onto another coordinate system); normalization (to ensure the same measurement unit and also to limit the energy of the signals in some perspectives); interpolation and resampling (to deal with missing data, irregular sampling rate, and to alleviate the mismatch between requested and effective sampling rate); dataset imbalance strategies (dealing with class and user imbalance); outlier removal; relabeling (when labels are reassigned to better match transitions between activities); trimming (when part of the signal is removed for some reason); separation (when the signal is separated into body and gravitational components), and arbitrary transformation (for early stage feature extraction)^1^.

We first focused on having dataset views as close as possible to the original datasets to verify whether our results would still corroborate previous findings in the literature. Hence, our approach to the preprocessing stage followed the principle of “minimum interference”. We call these versions of the datasets as the *baseline views*.

The adopted procedure involved processing the accelerometer (Acc) and gyroscope (Gyr) data by considering the sampling rate specified by the dataset authors and segmenting the raw time series into non-overlapping 3-second time windows. For the WISDM dataset, we opted to interpolate the signals in the baseline view to account for the trimodal distribution of the instantaneous sampling period. Given that both Acc and Gyr are tri-axial time series, the dimensionality of a baseline view sample is defined as 2 × 3 × sampling rate × 3 seconds, where the x-y-z Acc time series are concatenated with the x-y-z Gyr time series. The resulting baseline views were subsequently relabeled using a standard activity code and divided into training, validation, and test sets, ensuring balanced class distributions.

The partitioning process was executed at the user level with a 70/20/10 ratio for training, validation, and test sets, respectively. This means that for each dataset, users were partitioned into these three sets such that all samples from a given user were contained within a single set, thereby preventing any mixing of samples from the same user across different sets. This approach ensures that the model is tested on entirely unseen users, which better reflects real-world scenarios for human activity recognition (HAR) tasks. However, as users may have different numbers of samples, the actual number of samples in each set might not strictly follow the 70/20/10 ratio. After partitioning, the number of samples per activity within each set was balanced by randomly sampling the same number of samples for each activity, corresponding to the activity with the fewest samples in the set. It is important to highlight that our methodology was applied independently to each dataset, which may lead to variations in the number of samples across different partitions for each dataset. The number of samples in each dataset partition is detailed in Table 4.

**Table 4:** Number of samples for each dataset partition in the baseline views.

Dataset	Train	Validation	Test
KH	1386	426	144
MS	3558	420	1062
RW-T	8400	1764	2628
RW-W	10332	1854	2592
UCI	2420	340	690
WDM	8736	944	2596

**Fig. 1:**
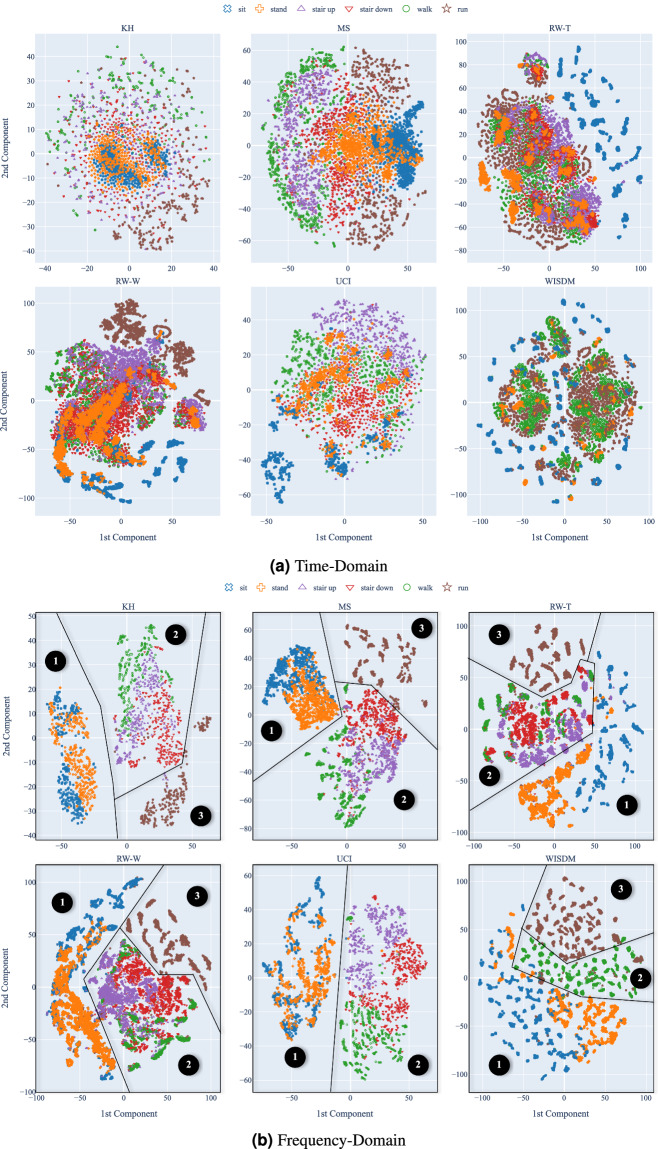
*t*-SNE visualization of the baseline views of the datasets.

Figure 1 shows the *t*-SNE visualization of the baseline views of the datasets in the time domain (1a) and in the frequency domain (1b), a common practice in HAR literature^30,31^. The *t*-SNE algorithm is a dimensionality reduction technique that preserves the local structure of data in a lower-dimensional space, making it easier to visualize the data distribution. In these figures, *t*-SNE was applied separately to each dataset, and the points are colored according to the activity label.

As we can notice, the classes within the datasets are better separated in the frequency domain than the time domain. This observation aligns with the expectation since the frequency domain often provides a more discriminative feature space for HAR tasks^23,32,33^. In the frequency domain, we can see that low-energy activities (➊) are well-separated from mid-energy activities (➋) and high-energy activities (➌). In the time domain, this separation is less clear despite the fact that the low-energy activities overlap in the same region.

We also assess the separability of the classes in the baseline view datasets by training different classifiers on each dataset and measuring their performances. We select three classical yet powerful classifiers: K-Nearest Neighbors (KNN), Random Forest (RF), and Support-Vector Machine (SVM). Additionally, we include twelve state-of-the-art deep learning models, each representing different principles, architectures, and complexities. These include two multi-layer perceptrons with 2 and 3 hidden layers (MLP-2L and MLP-3L), nine convolutional-based neural networks^12–15,34^, and one transformer-based network^14^. The training partition was used to train the classifiers, while the test partition was used to evaluate model performance. For the deep learning models, we used a validation partition to prevent overfitting and employed an early stopping criterion based on the validation loss.

The performance results are presented in Table 5. Since the baseline view is balanced regarding the number of samples per activity, we report the mean accuracy over five runs for each dataset and model. We explored two representations, the time domain (raw data) and the frequency domain, to evaluate model performance. To avoid introducing biases, we did not apply any feature extraction methods to the raw data, such as statistical features^33^. Moreover, it is expected that deep learning methods can learn proper features from the raw data without the need for additional feature extraction.

**Table 5:** Performance of models using baseline view.

Model	Time							Frequency						
	KH	MS	RW-T	RW-W	UCI	WDM	Mean	KH	MS	RW-T	RW-W	UCI	WDM	Mean
KNN	42.4%	81.0%	31.0%	46.1%	54.6%	60.4%	52.6%	86.8%	88.9%	73.6%	68.9%	81.7%	93.5%	82.2%
Random Forest	**80.6%**	87.9%	74.1%	77.4%	85.5%	92.1%	82.9%	79.7%	91.0%	83.8%	76.2%	92.2%	97.0%	86.7%
SVM	57.6%	76.9%	**81.1%**	68.8%	85.7%	92.5%	77.1%	70.1%	84.7%	85.5%	79.9%	86.2%	**98.6%**	84.2%
CNN (1D)^12^	77.5%	93.4%	80.8%	73.0%	95.2%	**95.6%**	**85.9%**	75.0%	91.2%	82.4%	82.9%	94.5%	96.6%	87.1%
CNN (2D)^12^	76.0%	93.2%	71.3%	**77.7%**	95.3%	91.5%	84.1%	79.6%	91.8%	79.6%	78.6%	89.1%	95.5%	85.7%
CNN PF^34^	79.4%	93.2%	70.7%	73.8%	94.2%	88.1%	83.3%	82.1%	92.1%	77.5%	83.2%	92.9%	96.6%	87.4%
CNN PFF^34^	79.4%	**93.5%**	73.4%	72.6%	95.8%	89.7%	84.1%	85.0%	90.4%	78.5%	**83.3%**	93.2%	96.3%	87.8%
ConvNet^13^	75.0%	93.5%	68.3%	74.2%	91.9%	90.5%	82.2%	**87.6%**	91.5%	**86.3%**	82.3%	**95.0%**	96.9%	**89.9%**
IMU CNN^14^	75.0%	87.4%	60.0%	64.3%	89.6%	84.8%	76.8%	84.2%	91.7%	75.6%	78.7%	94.1%	96.6%	86.8%
IMU Transf.^14^	74.9%	70.5%	73.0%	74.2%	92.2%	89.4%	79.0%	72.2%	73.1%	78.5%	76.9%	78.8%	96.3%	79.3%
MLP (2 Layers)	75.0%	83.2%	77.8%	63.3%	79.9%	91.4%	78.4%	86.7%	**92.7%**	82.5%	77.5%	92.8%	97.9%	88.4%
MLP (3 layers)	78.8%	82.7%	76.4%	64.5%	80.8%	88.6%	78.6%	86.2%	90.5%	81.7%	76.9%	93.7%	98.5%	87.9%
ResNet^15^	79.6%	86.8%	74.6%	76.9%	**97.6%**	91.9%	84.6%	70.4%	86.0%	80.5%	71.4%	92.8%	93.6%	82.5%
ResNetSE^67^	78.2%	90.9%	72.2%	76.1%	97.4%	92.9%	84.6%	76.2%	82.7%	80.3%	76.1%	91.6%	94.1%	83.5%
ResNetSE-5^67^	78.6%	89.0%	70.0%	75.3%	95.3%	90.4%	83.1%	76.1%	90.3%	79.0%	78.2%	92.5%	94.5%	85.1%
Max	80.6%	93.5%	81.1%	77.7%	97.6%	95.6%	85.9%	87.6%	92.7%	86.3%	83.3%	95.0%	98.6%	89.9%

The best results for each dataset and for each domain (time and frequency) are highlighted in bold. **Mean** column represents the average performance of the model in the datasets.

Firstly, it is worth remarking that the performances of the models vary significantly across the datasets. The CNN (1D)^12^ model stands out, achieving the highest average performance. It also performs well in the frequency domain, with a mean accuracy close to the best model. Additionally, all deep learning models perform well in both domains. Finally, the results reported in Table 5 demonstrate that although time-domain data may not be as informative as frequency-domain data in this task, it is still possible to achieve good results using machine/deep learning models.

### Standardization process

The baseline views of the datasets differ in format, including dimensionality, accelerometer units of measurement, and sampling rate. While all datasets are sliced into 3-second time windows, relabeled, and split into training and test sets, allowing isolated evaluation of model performance, these differences in data format prevent direct comparison. To address this issue, we propose a new view of the datasets, called the **standardized view**, to ensure uniform representation across all datasets.

In this standardized view, we apply a series of preprocessing steps to standardize the datasets, as detailed in Table 6. It is important to emphasize that this standardization process does not change the number of samples in each dataset partition but only the number of features per sample.

**Table 6:** Set of preprocessing steps applied to generate the Standardized view for each dataset.

Preprocessing Step	KH	MSa	RW	UCI	WISDM
$G\to m/{s}^{2}$		✓		✓	
Resampling (20 Hz)	✓	✓	✓	✓	✓
Gravity Removal		✓	✓	✓	✓
Slicing (3 s)	✓	✓	✓	✓	✓
Re-labeling	✓	✓	✓	✓	✓
Train/Test split	✓	✓	✓	✓	✓

*a*: MotionSense does not provide total acceleration data, only the body and gravity components. To keep it consistent with other datasets, we combined both components together and removed the gravity acceleration by applying the Butterworth filter.

Firstly, we convert the accelerometer measurement unit from *G* to *m*/*s*^2^ for the MS and UCI datasets. We then resample the data to 20 Hz and remove gravity acceleration by applying a high-pass 3rd order Butterworth filter with a cutoff frequency of 0.3 Hz. Next, we split the data into non-overlapping 3-second time windows and relabel the data to ensure activities are consistently encoded across datasets. Finally, we split the samples into training, validation, and test sets, ensuring different users are in the training and test sets and maintaining the same procedure as the baseline views. The standardized views use the same Train/Validation/Test split employed in the baseline views to preserve the same samples in the training, validation, and test subsets.

The only differences from the baseline views are the initial three preprocessing steps necessary to ensure all datasets are compatible with the data format. The final three steps remain consistent with the baseline view. This view corresponds to our DAGHAR benchmark dataset^21^.

**Fig. 2:**
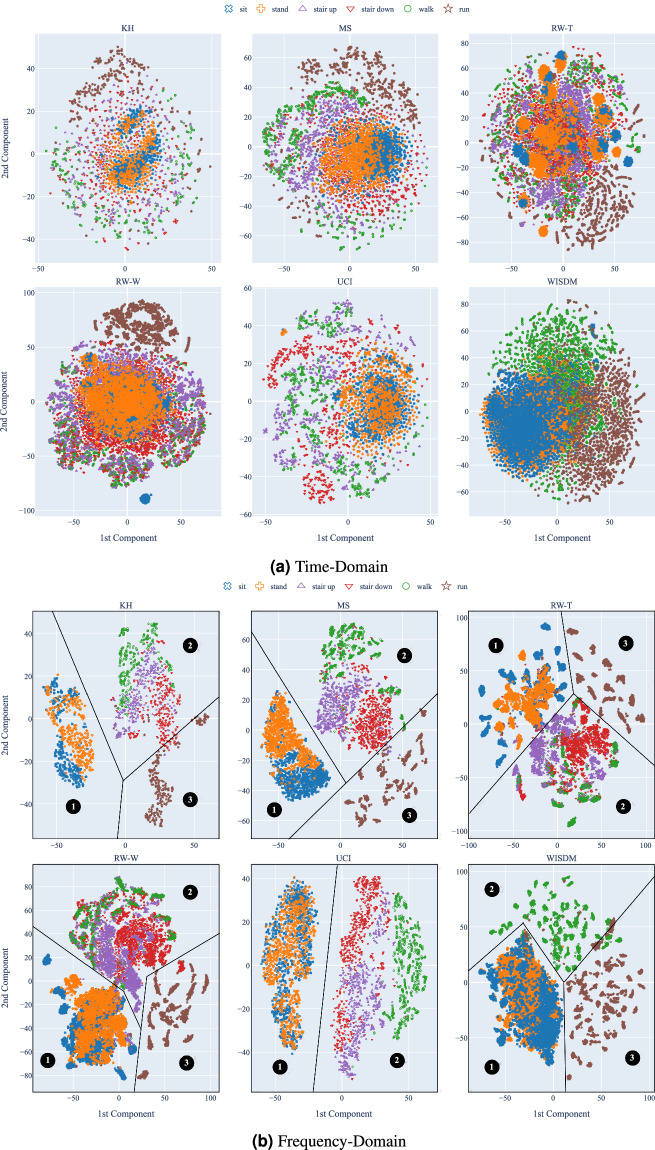
*t*-SNE visualization of the standardized views of the datasets.

Figure 2 shows the *t*-SNE visualization of the standardized views of the datasets in the time domain (2a) and in the frequency domain (2b). The goal is to demonstrate that the standardization process preserves underlying data patterns while making datasets more comparable. In the frequency domain, classes are better separated than in the time domain, maintaining the same pattern observed with the baseline views. Low-energy (➊), mid-energy (➋), and high-energy (➌) activities forms three separated clusters. The time domain also shares the same pattern as the baseline views but with less class separation than the frequency domain.

This indicates that the standardization process preserves the intrinsic differences among the datasets while removing external biases that could impact conclusions about generalization. By factoring out these biases, we can evaluate the generalization capabilities of models in a more controlled manner.

Table 7 shows the performance of the models on the standardized views of the datasets. Analogously to the case with the baseline views, we can observe that convolutional neural networks are generally the best performing models: they reached the best performance in 10 out of the 12 scenarios (dataset + domain).

**Table 7:** Performance of models using standardized view.

Model	Time							Frequency						
	KH	MS	RW-T	RW-W	UCI	WDM	Mean	KH	MS	RW-T	RW-W	UCI	WDM	Mean
KNN	49.3%	66.6%	43.6%	53.4%	66.7%	60.7%	56.7%	86.8%	90.9%	65.2%	74.5%	81.7%	89.8%	81.5%
Random Forest	80.7%	89.1%	62.5%	67.2%	88.1%	85.5%	78.8%	82.6%	92.9%	80.5%	74.7%	92.8%	89.5%	85.5%
SVM	61.1%	76.7%	64.7%	65.2%	78.6%	74.6%	70.2%	71.5%	81.9%	74.1%	73.6%	79.7%	78.7%	76.6%
CNN (1D)^12^	78.1%	92.2%	69.8%	73.4%	94.9%	**90.3%**	83.1%	73.5%	91.1%	74.7%	81.6%	94.0%	90.7%	84.3%
CNN (2D)^12^	80.7%	94.2%	74.0%	75.8%	93.7%	87.6%	84.3%	77.2%	91.9%	74.6%	80.9%	91.0%	89.9%	84.3%
CNN PF^34^	79.2%	94.9%	69.9%	79.3%	95.2%	85.7%	84.0%	80.6%	90.7%	65.6%	83.8%	95.3%	91.0%	84.5%
CNN PFF^34^	80.0%	94.0%	67.4%	**80.6%**	**96.9%**	87.5%	**84.4%**	78.2%	91.7%	64.3%	83.0%	**95.7%**	90.2%	83.8%
ConvNet^13^	78.5%	**95.8%**	63.6%	77.5%	96.9%	87.3%	83.3%	81.2%	92.4%	**81.5%**	**84.5%**	94.4%	91.3%	**87.6%**
IMU CNN^14^	78.2%	87.7%	59.9%	69.7%	91.9%	83.6%	78.5%	80.7%	**93.6%**	65.3%	81.1%	95.5%	**91.7%**	84.6%
IMU Transf.^14^	73.5%	64.3%	63.0%	73.1%	62.8%	45.9%	63.8%	70.8%	77.6%	63.5%	77.4%	78.7%	57.9%	71.0%
MLP (2 Layers)	75.8%	84.3%	57.3%	62.5%	79.7%	81.7%	73.5%	**86.9%**	91.2%	74.3%	80.9%	92.6%	90.3%	86.0%
MLP (3 layers)	79.4%	84.1%	57.6%	64.1%	81.8%	81.4%	74.7%	86.0%	90.9%	74.8%	79.7%	93.5%	90.5%	85.9%
ResNet^15^	81.4%	79.5%	67.6%	74.6%	91.0%	79.6%	78.9%	71.9%	85.9%	67.2%	80.6%	90.7%	85.0%	80.2%
ResNetSE^67^	80.8%	83.0%	69.6%	74.7%	90.7%	76.9%	79.3%	67.4%	84.6%	70.0%	74.5%	84.6%	85.0%	77.7%
ResNetSE-5^67^	**82.6%**	84.9%	**74.1%**	69.1%	92.1%	82.0%	80.8%	71.3%	88.5%	68.6%	78.6%	91.3%	81.0%	79.9%
Max	82.6%	95.8%	74.1%	80.6%	96.9%	90.3%	84.4%	86.9%	93.6%	81.5%	84.5%	95.7%	91.7%	87.6%

The best results for each dataset and for each domain (time and frequency) are highlighted in bold. **Mean** column represents the average performance of the model in the datasets.

Table 8 shows the ratio of model performance using the standardized views compared to the baseline views, providing a more comprehensive assessment of the standardization process’s impact on model performance. It is worth recognizing that the standardized views slightly increase the classification difficulty in most cases, although the differences are not significant. This suggests that the standardization process does not eliminate the intrinsic differences among the datasets. However, it enables the evaluation of model generalization capabilities in cross-dataset scenarios, which is crucial for assessing the performance of HAR models in new, unseen environments, as described in the sequence.

**Table 8:** Ratio of model performance between the baseline view and the standardized view.

Model	Time							Frequency						
	KH	MS	RW-T	RW-W	UCI	WDM	Mean	KH	MS	RW-T	RW-W	UCI	WDM	Mean
KNN	1.16x	0.82x	1.41x	1.16x	1.22x	1.01x	1.08x	1.00x	1.02x	0.89x	1.08x	1.00x	0.96x	0.99x
Random Forest	1.00x	1.01x	0.84x	0.87x	1.03x	0.93x	0.95x	1.04x	1.02x	0.96x	0.98x	1.01x	0.92x	0.99x
SVM	1.06x	1.00x	0.80x	0.95x	0.92x	0.81x	0.91x	1.02x	0.97x	0.87x	0.92x	0.92x	0.80x	0.91x
CNN (1D)^12^	1.01x	0.99x	0.86x	1.01x	1.00x	0.95x	0.97x	0.98x	1.00x	0.91x	0.98x	1.00x	0.94x	0.97x
CNN (2D)^12^	1.06x	1.01x	1.04x	0.98x	0.98x	0.96x	1.00x	0.97x	1.00x	0.94x	1.03x	1.02x	0.94x	0.98x
CNN PF^34^	1.00x	1.02x	0.99x	1.07x	1.01x	0.97x	1.01x	0.98x	0.99x	0.85x	1.01x	1.03x	0.94x	0.97x
CNN PFF^34^	1.01x	1.01x	0.92x	1.11x	1.01x	0.98x	1.00x	0.92x	1.02x	0.82x	1.00x	1.03x	0.94x	0.96x
ConvNet^13^	1.05x	1.02x	0.93x	1.05x	1.06x	0.96x	1.01x	0.93x	1.01x	0.94x	1.03x	0.99x	0.94x	0.97x
IMU CNN^14^	1.04x	1.00x	1.00x	1.08x	1.03x	0.99x	1.02x	0.96x	1.02x	0.86x	1.03x	1.01x	0.95x	0.97x
IMU Transf.^14^	0.98x	0.91x	0.86x	0.99x	0.68x	0.51x	0.81x	0.98x	1.06x	0.81x	1.01x	1.00x	0.60x	0.90x
MLP (2 Layers)	1.01x	1.01x	0.74x	0.99x	1.00x	0.89x	0.94x	1.00x	0.98x	0.90x	1.04x	1.00x	0.92x	0.97x
MLP (3 layers)	1.01x	1.02x	0.75x	0.99x	1.01x	0.92x	0.95x	1.00x	1.01x	0.92x	1.04x	1.00x	0.92x	0.98x
ResNet^15^	1.02x	0.92x	0.91x	0.97x	0.93x	0.87x	0.93x	1.02x	1.00x	0.83x	1.13x	0.98x	0.91x	0.97x
ResNetSE^67^	1.03x	0.91x	0.97x	0.98x	0.93x	0.83x	0.94x	0.88x	1.02x	0.87x	0.98x	0.92x	0.90x	0.93x
ResNetSE-5^67^	1.05x	0.95x	1.06x	0.92x	0.97x	0.91x	0.97x	0.94x	0.98x	0.87x	1.00x	0.99x	0.86x	0.94x
Max (Ratio)	1.03x	1.02x	0.91x	1.04x	0.99x	0.95x	0.98x	0.99x	1.01x	0.94x	1.01x	1.01x	0.93x	0.97x

Values close to one indicate no significant difference between the two views, values below one indicate better performance in the baseline view, and values above one indicate better performance in the standardized view. The Max (Ratio) line is the ratio between both maximum values of the two views.

## Data Records

The DAGHAR benchmark dataset is available at Zenodo repository^21^. Each dataset view is stored in a separate folder: baseline_view and standardized_view. Inside each view folder, there are subfolders corresponding to each dataset: KuHar, MotionSense, RealWorld-Thigh, RealWorld-Waist, UCI, and WISDM. Each dataset folder contains the following three CSV files, corresponding to each partition of the dataset: train.csv, validation.csv, and test.csv.

For each CSV file, the rows correspond to the samples, and the columns correspond to the features. Thus, each row stores the time-series data of a 3-second window from both triaxial accelerometer and gyroscope sensors. The following columns are always present in the CSV files:

- accel-x-: Columns starting with this prefix correspond to instants of the x-axis accelerometer time series. For instance, accel-x-0 is associated with the first instant of the x-axis Acc time series, while accel-x-1 corresponds to the second instant of observation. Thus, a 3-second window sampled at 20 Hz will have 60 columns for the x-axis accelerometer time series. The same logic applies to all other prefixes.
- accel-y-: Columns starting with this prefix correspond to an instant of the y-axis Acc time series.
- accel-z-: Columns starting with this prefix correspond to an instant of the z-axis Acc time series.
- gyro-x-: Columns starting with this prefix correspond to an instant of the x-axis Gyr time series.
- gyro-y-: Columns starting with this prefix correspond to an instant of the y-axis Gyr time series.
- gyro-z-: Columns starting with this prefix correspond to an instant of the z-axis Gyr time series.
- standard activity code: The activity label of the sample matches the same code across all datasets.

Some datasets may also have metadata information, which is dataset-specific and can be discarded for training and evaluation purposes. However, we maintain these metadata in the same CSV in case it is needed.

## Technical Validation

**Fig. 3:**
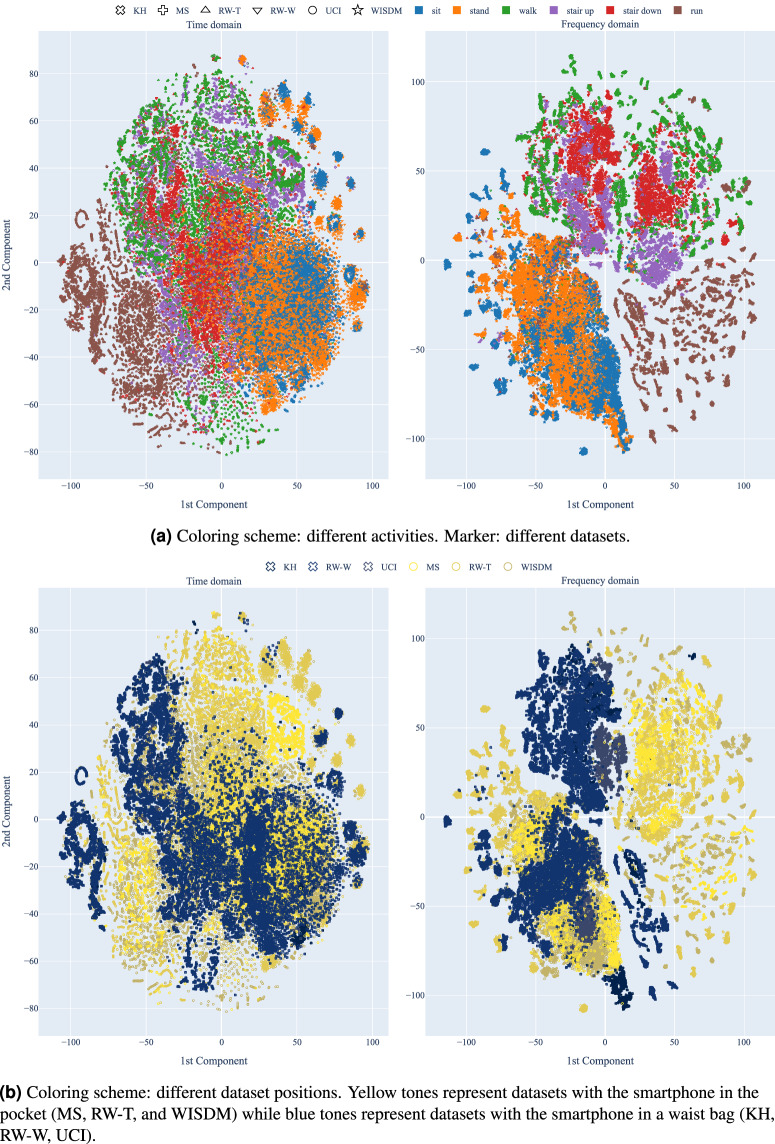
*t*-SNE visualization of the standardized views of the datasets. The *t*-SNE was applyied to all datasets together and the coloring/marker scheme is based on the activity label (3a) and the smartphone position (3b). Thus, both figures show the same data distribution, but with different coloring/marker schemes.

Using the standardized views, DAGHAR^21^ enables the evaluation of model generalization capabilities in cross-dataset scenarios, factoring out known biases and ensuring a more controlled environment. Figure 3 shows the *t*-SNE visualization with all samples from all datasets concatenated into a single dataset and projected into a single 2D space. The coloring and marker scheme is based on the activity label (3a) and the smartphone position (3b). This visualization allows us to observe the data distribution across all datasets, providing insights into their similarities and differences.

By examining Fig. 3a, in the frequency domain, we can see that the classes are well-separated, with low-energy, mid-energy, and high-energy activities forming distinct clusters, independent of the dataset. This corroborates the insights on individual datasets presented in previous sections. In the time domain, however, the classes are less separated, with some overlap between low-energy and other activities.

An interesting point to highlight is associated with the smartphone position (Fig. 3b), which should be analyzed in conjunction with the activity label (Fig. 3a). In the frequency domain, datasets with different smartphone positions (pocket and waist bag) occupy the same space for low-energy activities, indicating that smartphone position does not significantly impact the data distribution for these activities. However, for other activities, datasets with different smartphone positions form distinct clusters, suggesting that smartphone position may significantly impact the data distribution for these activities. For instance, activities like walking, walking upstairs, and walking downstairs seem more affected by the smartphone position, as they are more sensitive to the body’s movement and position. The same trend appears in the time domain but with less cluster separation.

This suggests a domain shift between datasets with different smartphone positions, which could impact model generalization capabilities in cross-dataset scenarios. Therefore, smartphone position should be considered in domain adaptation or domain generalization studies to ensure robust performance.

An usual procedure to estimate the domain shift resorts to the Maximum Mean Discrepancy (MMD) metric^35^. MMD is a statistical measure of the discrepancy between two probability distributions. A low MMD value (close to 0) suggests that the distributions of the two datasets are very similar, while a high MMD value indicates significant differences between the distributions. These differences can arise due to variations in mean, variance, or other higher-order moments.

**Fig. 4:**
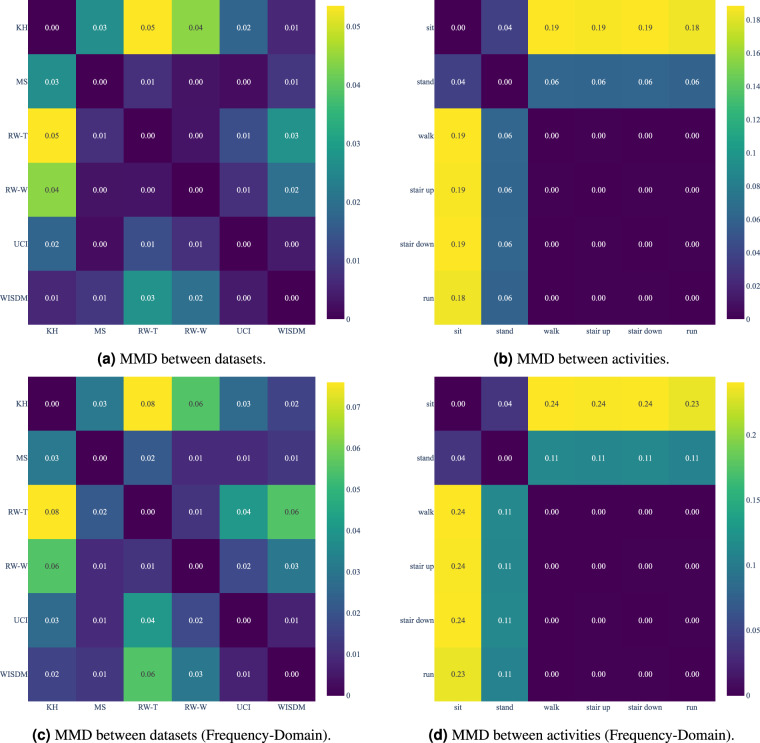
Mean Maximum Discrepancy (MMD) between datasets and activities in the standardized views.

Figure 4 presents two MMD matrices: one showing the MMD between all pairs of datasets in the standardized views independent of the activities, and the other showing the MMD between all pairs of activities in the standardized views, disregarding the dataset. The diagonal values are always 0 because they represent the MMD of a dataset with itself. In contrast, the off-diagonal values represent the MMD between two different datasets (MMD generates a symmetric matrix as the order of the datasets does not matter). For both matrices, we calculated MMD using a Gaussian kernel with γ = 1.0.

The MMD matrix between datasets reveals that KH is one of the most dissimilar datasets compared to the others (MS, RW-T, RW-W), as indicated by higher MMD values. This is intriguing since KH has the same smartphone position as the UCI and RW-W datasets. Similar but less pronounced patterns are observed with other datasets, such as MS.

The MMD matrix between activities shows that “sit” and “stand” are the most dissimilar activities compared to the others, indicated by higher MMD values. This quantitatively corroborates the insights from *t*-SNE visualizations, where these activities are separated from others. The mid-energy and high-energy activities are more similar, as evidenced by lower MMD values, indicating a greater similarity in their data distributions despite being different activities.

Our evaluation of the models on the standardized views of the datasets in a cross-dataset scenario is based on a leave-one-dataset-out strategy. This widely used method for assessing domain adaptation and generalization involves training a model on all datasets except one, and then testing it on the left-out dataset. The reported value is the performance on the left-out dataset. We repeat this process for each dataset to evaluate the generalization capabilities of the models across different datasets.

The results are presented in Table 9, and the performance improvement compared to the same-dataset regime is shown in Table 10. Only training subsets are used for training the models, and only test subsets are used for evaluation. This ensures that the models are not exposed to any test data during training, preventing data leakage and ensuring a fair comparison between models trained in other regimes.

**Table 9:** Performance of models using standardized view in the cross-dataset scenario, using leave-one-dataset-out strategy.

Model	Time							Frequency						
	KH	MS	RW-T	RW-W	UCI	WDM	Mean	KH	MS	RW-T	RW-W	UCI	WDM	Mean
KNN	58.3%	49.8%	38.1%	36.6%	34.6%	43.4%	43.5%	61.1%	81.4%	66.4%	58.8%	59.6%	72.2%	66.6%
Random Forest	54.3%	60.3%	47.6%	42.6%	65.8%	59.0%	54.9%	62.8%	82.8%	67.0%	69.6%	79.5%	71.4%	72.2%
SVM	52.1%	62.4%	49.5%	49.9%	66.5%	55.7%	56.0%	52.8%	80.6%	72.4%	68.3%	67.7%	69.6%	68.6%
CNN (1D)^12^	63.3%	**79.0%**	**71.7%**	68.0%	**81.1%**	**70.3%**	**72.2%**	66.2%	84.6%	71.4%	69.7%	77.2%	73.9%	73.9%
CNN (2D)^12^	61.4%	70.2%	61.9%	67.8%	70.7%	60.3%	65.4%	66.1%	83.6%	73.9%	69.9%	75.9%	73.3%	73.8%
CNN PF^34^	61.9%	67.2%	65.0%	66.1%	73.0%	54.9%	64.7%	71.9%	82.8%	70.5%	70.6%	78.3%	74.1%	74.7%
CNN PFF^34^	63.2%	66.3%	64.8%	67.5%	74.4%	56.0%	65.4%	70.3%	84.3%	69.5%	70.4%	77.6%	74.1%	74.4%
ConvNet^13^	**63.9%**	65.6%	47.3%	61.2%	70.1%	53.9%	60.3%	69.7%	**85.6%**	**75.0%**	**71.3%**	**81.8%**	**79.1%**	**77.1%**
IMU CNN^14^	54.2%	62.5%	42.9%	48.7%	64.8%	59.4%	55.4%	70.4%	85.6%	68.5%	71.3%	78.8%	74.7%	74.9%
IMU Transf.^14^	63.1%	58.5%	35.7%	57.5%	62.6%	59.8%	56.2%	67.5%	84.0%	67.7%	68.1%	76.4%	73.8%	72.9%
MLP (2 Layers)	55.7%	71.8%	54.9%	55.1%	68.7%	60.9%	61.2%	74.2%	83.9%	70.5%	65.0%	74.6%	73.2%	73.5%
MLP (3 layers)	53.3%	73.6%	54.2%	56.3%	67.3%	59.7%	60.8%	**77.9%**	85.4%	73.5%	67.2%	75.5%	75.2%	75.8%
ResNet^15^	58.5%	68.0%	41.1%	66.9%	76.8%	57.4%	61.4%	62.9%	79.8%	66.8%	65.5%	74.1%	69.1%	69.7%
ResNetSE^67^	60.4%	68.8%	47.1%	**68.1%**	73.3%	54.2%	62.0%	58.2%	77.5%	68.4%	66.9%	74.7%	67.1%	68.8%
ResNetSE-5^67^	49.0%	67.0%	49.6%	66.2%	72.7%	51.7%	59.4%	65.4%	81.1%	67.5%	66.3%	75.0%	70.6%	71.0%
Max	63.9%	79.0%	71.7%	68.1%	81.1%	70.3%	72.2%	77.9%	85.6%	75.0%	71.3%	81.8%	79.1%	77.1%

The best results for each dataset and for each domain (time and frequency) are highlighted in bold. **Mean** column represents the average performance of the model in the datasets.

**Table 10:** Ratio of model performance between standard training and cross-dataset training using a leave-one-dataset-out strategy.

Model	Time							Frequency						
	KH	MS	RW-T	RW-W	UCI	WDM	Mean	KH	MS	RW-T	RW-W	UCI	WDM	Mean
KNN	1.18x	0.75x	0.87x	0.69x	0.52x	0.72x	0.77x	0.70x	0.90x	1.02x	0.79x	0.73x	0.80x	0.82x
Random Forest	0.67x	0.68x	0.76x	0.63x	0.75x	0.69x	0.70x	0.76x	0.89x	0.83x	0.93x	0.86x	0.80x	0.84x
SVM	0.85x	0.81x	0.77x	0.76x	0.85x	0.75x	0.80x	0.74x	0.98x	0.98x	0.93x	0.85x	0.88x	0.90x
CNN (1D)^12^	0.81x	0.86x	1.03x	0.93x	0.85x	0.78x	0.87x	0.90x	0.93x	0.96x	0.85x	0.82x	0.82x	0.88x
CNN (2D)^12^	0.76x	0.75x	0.84x	0.89x	0.75x	0.69x	0.78x	0.86x	0.91x	0.99x	0.86x	0.83x	0.82x	0.88x
CNN PF^34^	0.78x	0.71x	0.93x	0.83x	0.77x	0.64x	0.77x	0.89x	0.91x	1.08x	0.84x	0.82x	0.81x	0.88x
CNN PFF^34^	0.79x	0.71x	0.96x	0.84x	0.77x	0.64x	0.77x	0.90x	0.92x	1.08x	0.85x	0.81x	0.82x	0.89x
ConvNet^13^	0.81x	0.68x	0.74x	0.79x	0.72x	0.62x	0.72x	0.86x	0.93x	0.92x	0.84x	0.87x	0.87x	0.88x
IMU CNN^14^	0.69x	0.71x	0.72x	0.70x	0.71x	0.71x	0.71x	0.87x	0.92x	1.05x	0.88x	0.83x	0.82x	0.89x
IMU Transf.^14^	0.86x	0.91x	0.57x	0.79x	1.00x	1.30x	0.88x	0.95x	1.08x	1.07x	0.88x	0.97x	1.27x	1.03x
MLP (2 Layers)	0.73x	0.85x	0.96x	0.88x	0.86x	0.75x	0.83x	0.85x	0.92x	0.95x	0.80x	0.81x	0.81x	0.85x
MLP (3 layers)	0.67x	0.88x	0.94x	0.88x	0.82x	0.73x	0.81x	0.91x	0.94x	0.98x	0.84x	0.81x	0.83x	0.88x
ResNet^15^	0.72x	0.85x	0.61x	0.90x	0.84x	0.72x	0.78x	0.87x	0.93x	0.99x	0.81x	0.82x	0.81x	0.87x
ResNetSE^67^	0.75x	0.83x	0.68x	0.91x	0.81x	0.70x	0.78x	0.86x	0.92x	0.98x	0.90x	0.88x	0.79x	0.89x
ResNetSE-5^67^	0.59x	0.79x	0.67x	0.96x	0.79x	0.63x	0.73x	0.92x	0.92x	0.98x	0.84x	0.82x	0.87x	0.89x
Max (Ratio)	0.77x	0.82x	0.97x	0.85x	0.84x	0.78x	0.86x	0.90x	0.92x	0.92x	0.84x	0.86x	0.86x	0.88x

Values close to one indicate no significant difference between the two scenarios, values below one indicate better performance in the standard training scenario, and values above one indicate better performance in the cross-dataset scenario. The Max (Ratio) line is the ratio between both maximum values of the two scenarios.

A significant drop in performance can be observed when comparing cross-dataset evaluation to same-dataset evaluation. This is expected, as cross-dataset evaluation is more challenging since the model must generalize to new, unseen environments. Interestingly, datasets with similar smartphone positions and low MMD values between them also exhibit a significant drop in performance. This indicates that even when a model is exposed to similar environments or is made more generic by incorporating more samples from different environments, it still needs to generalize to new scenarios. This suggests that external factors such as user demographics, data collection protocols, and other variables may significantly impact model performance more than smartphone position alone, highlighting the necessity of research into adaptation strategies.

Interestingly, there results reveal that convolutional models consistently outperforms other models in cross-dataset evaluation, demonstrating its robustness to domain shifts. This novel insight underscores the potential of convolutional-based models in generalizing to new, unseen environments, making them a promising option for HAR tasks.

## Usage Notes

In this section, we demonstrate how to read and handle the data, train and evaluate machine learning models, as well as how to extend the DAGHAR dataset using the same standardization process.

### Reading data and training machine learning models

The dataset consists of time series data from accelerometers and gyroscopes, along with associated metadata. These are stored in a single CSV file per partition, as described in the Data Records section. The data can be easily handled using the Pandas library in Python to train machine learning models.

Below is a simple example that shows how to read the data, train a Random Forest model from the scikit-learn library, and evaluate it on a test set. The trainable data, which is represented as an (*N*, *F*) matrix, where *N* is the number of samples and *F* is the number of features (calculated as 2 × 3 × sampling rate × 3 seconds), is stored in a variable named X, while the labels are stored in a variable named y, which is a common practice in machine learning tasks.


import pandas as pdfrom sklearn.ensemble import RandomForestClassifier# Global variables, adjust accordinglysampling_rate_hz = 20window_size_seconds = 3num_features_per_sensor_axis = sampling_rate_hz * window_size_seconds# Load train and test datatrain_data = pd.read_csv('path/to/train.csv') test_data = pd.read_csv('path/to/test.csv')# Create a list of column names to select for the X matrixX_columns_to_select = []for axis in ['accel-x', 'accel-y', 'accel-z', 'gyro-x', 'gyro-y', 'gyro-z']:for i in range(num_features_per_sensor_axis):X_columns_to_select.append(f'{axis}-{i}')# Name of label columny_column = 'standard activity code'# Select the columns to compose the train X matrixX_train = train_data[X_columns_to_select].valuesy_train = train_data[y_column].values# Select the columns to compose the test X matrixX_test = test_data[X_columns_to_select].valuesy_test = test_data[y_column].values# Create the RF model, train it, and evaluate accuracy on the test setmodel = RandomForestClassifier()model.fit(X_train, y_train)# Predict and calculate mean accuracyaccuracy = model.score(X_test, y_test)print(f'Accuracy: {accuracy}')


For training deep learning models, we recommend using the PyTorch Lightning library. Additionally, we recommend using the Minerva framework (https://github.com/discovery-unicamp/Minerva), which is built on top of PyTorch Lightning and offers a set of models, data modules, and tools for training deep learning models and evaluating them on the DAGHAR dataset as well as other HAR datasets.

### Extending DAGHAR

Datasets often come in various formats and structures, which can make the standardization process challenging. To address this, we have organized our processing scripts into two parts, available at official Github repository: reading datasets and standardizing them. The first part is the most complex, as it requires understanding the dataset’s structure and determining the proper way to read it. The second part is more straightforward since the standardization process is almost uniform across all datasets and the same steps can be applied to most datasets, with minor adjustments.

To add a new dataset, the user must create a function at readers.py file, whose name is prefixed with read_, that reads the dataset given a valid path and returns a Pandas DataFrame with some required information. This dataframe is what we will name as the “intermediate representation” of the dataset, that is a single table where each row corresponds to a time step with its respective features. All of our processing steps will be applied to this intermediate representation to standardize the dataset. The **required** columns are as follows:

- accel-x, accel-y, accel-z: accelerometer data;
- gyro-x, gyro-y, gyro-z: gyroscope data;
- accel-start-time, accel-end-time: the start and end times of the accelerometer data (for a single time instant, start and end times are the same);
- gyro-start-time, gyro-end-time: the start and end times of the gyroscope data (for a single time instant, start and end times are the same);
- activity code: the activity code associated with the time instant;
- user: the user associated with the time instant;
- trial: the trial associated with the time instant, as one user may have multiple trials;
- index: the index of the time instant within a user’s trial; and
- csv: the CSV file name of the dataset.

The dataframe may also contain other metadata information (additional columns, along the required ones), which is dataset-specific and can be discarded (or used) during the standardization process.

After creating the function, the user must define a pipeline to standardize the dataset. A pipeline is a sequence of steps applied sequentially to the dataset. Steps are simple functions that take a Pandas DataFrame as input and return a modified Pandas DataFrame as output. Each step is responsible for a specific operation, such as applying a Butterworth filter or performing normalization. Since datasets can vary significantly in characteristics, different steps may be needed to standardize them. Thus, the user must define a pipeline (inside pipelines.py file) that is specific to the dataset being processed, by creating a list of steps that will be applied to the intermediate representation. Common steps are already implemented, and users are encouraged to reuse them when applicable.

For more detailed instructions on how to add a new dataset, please refer to the official repository.

## Data Availability

All codes are open-source, licensed under MIT License, and available at https://github.com/H-IAAC/DAGHAR.
